# Development of pyroptosis inhibitors for Alzheimer’s disease

**DOI:** 10.1002/alz.085249

**Published:** 2025-01-09

**Authors:** Yiming Xu, Savannah Biby, Shijun Zhang

**Affiliations:** ^1^ Virginia Commonwealth University, Richmond, VA USA

## Abstract

**Background:**

Pyroptosis is a type of inflammasome‐dependent cell death, in which gasdermin D (GSDMD) plays key roles as the executor. Neuroinflammation and pyroptosis have been indicated critical roles in neurodegenerative disorders including Alzheimer’s disease (AD). Therefore, novel GSDMD inhibitors represent valuable probes to understand and validate GSDMD as a viable drug target for AD.

**Method:**

Biophysical screening and rational design were employed to design novel GSDMD inhibitors. Cellular testing using iBMDMs to examine membrane pore formation, LDH and Il‐1 release, and GSDMD cleavage.

**Result:**

A series compounds based on a scaffold identified via biophysical screening against recombinant mouse GSDMD were designed and synthesized to optimize the potency and selectivity. Among the tested compounds, several lead compounds were identified with nanomolar binding affinity to GSDMD and in vitro GSDMD and pyroptosis inhibition.

**Conclusion:**

Novel GSDMD inhibitors were successfully identified and functional evaluation demonstrated promising and selective inhibition on GSDMD. The results encourage further characterization of the lead compounds as novel probes.

